# Incidence, risk factors, and quality of life of low back pain after cesarean delivery and vaginal delivery at Dilla University General Hospital: a prospective cohort study

**DOI:** 10.3389/fmed.2025.1495335

**Published:** 2025-05-21

**Authors:** Muhiddin Tadesse Barega, Yadasa Hemba, Abdulfeta Redela, Timsel Girma, Mesay Milkiyas Wonte, Ali Dimma, Sofia Assen, Shamil Eanga

**Affiliations:** 1 Department of Anesthesiology, College of Medicine and Health Sciences, Dilla University, Dilla, Ethiopia; 2 Department of Anesthesiology, College of Medicine and Health Sciences, Wolkite University, Wolkite, Ethiopia; 3 Department of Obstetrics and Gynecology, College of Medicine and Health Sciences, Dilla University, Dilla, Ethiopia

**Keywords:** low back pain, postpartum low back pain, quality life, risk factors, Ethiopia

## Abstract

**Introduction:**

Low back pain after delivery is a common and often debilitating condition that is frequently underdiagnosed and poorly managed. It is defined as discomfort or stiffness in the lower back. This study aimed to assess the incidence, risk factors, and quality of life associated with low back pain following vaginal and cesarean deliveries at Dilla University General Hospital in South Ethiopia from November 2021 to November 2022.

**Methods:**

A prospective cohort study was conducted on 129 pregnant mothers at Dilla University General Hospital. Demographic data and obstetric history were recorded before delivery. Postpartum data on the presence and severity of back pain were collected at multiple intervals from the first 24 h up to 6 months. The severity of back pain and quality of life were assessed by a numerical rating scale and the Short Form-36 health-related quality of life survey, respectively. Risk factors for postpartum low back pain were identified as significant at *p* < 0.05.

**Results:**

The incidence of low back pain was significantly higher in the cesarean delivery group compared to the vaginal delivery group from the second postpartum day to the fourth week (*p* < 0.05), but there were no significant differences between the groups on the first postpartum day or after a month. Most participants in both groups reported mild low back pain during follow-up. Body mass index over 30 kg/m^2^ [AOR = 3.01 (1.92–5.43), *p* = 0.013] and post-term gestation [AOR = 1.79 (1.23–7.75), *p* = 0.025] were identified as risk factors. Mothers who delivered via spontaneous vaginal delivery had a higher quality of life score (79.13 ± 7.06) compared to those who had a cesarean delivery (73.12 ± 3.46), with a *p*-value of 0.006 and an effect size of 0.48.

**Conclusion:**

Cesarean delivery is linked to a higher incidence of postpartum low back pain compared to spontaneous vaginal delivery from the second day to the fourth week after childbirth. A higher body mass index and post-term gestation were identified as risk factors. Additionally, the impact of low back pain on the quality of life accentuates the need for comprehensive postpartum care.

## Introduction

Low back pain (LBP) is defined as discomfort or stiffness localized in the lower back. It is a prevalent condition after delivery and can be debilitating, with symptoms sometimes extending to the legs (1, 2). Low back pain in postpartum mothers presents unique challenges, particularly in the first weeks to a year after childbirth. During this time, women experience significant physical and emotional adjustments. Postpartum low back pain refers to LBP occurring within the first six weeks to a year after childbirth (1–3).

Existing literature reports the prevalence of postpartum low back pain to range from 2 to 75%, with many cases potentially representing a continuation of back pain experienced during pregnancy into the postpartum period (3–5). Despite its prevalence, postpartum low back pain is often underdiagnosed and inadequately managed. It is commonly misunderstood as a natural consequence of childbirth that will resolve on its own. However, for many women, this pain persists for months or even years, potentially leading to chronic pain and disability (4–8).

Persistent pain can interfere with a mother's ability to carry out daily activities, such as household chores, self-care, and the physical demands of infant care, including feeding, lifting, and carrying (9). In addition to physical limitations, low back pain after delivery can also lead to emotional challenges, including increased stress, anxiety, and even postpartum depression (10). This pain may hinder a mother's ability to fully engage in bonding with her newborn, impact her relationships with family members, and delay her return to work or normal daily activities. As a result, it diminishes her overall quality of life during a critical adjustment period. This highlights the need for more focused postpartum care to address low back pain (LBP) and its lasting impact (11–14).

The risk factors for postpartum low back pain are multifactorial, with both maternal and obstetric elements playing significant roles. These factors include the mode of delivery, with cesarean section delivery and vaginal delivery presenting different risks (6, 15). Musculoskeletal strain during pregnancy, including shifting body weight and changes in posture, as well as pre-existing conditions such as a history of back pain, are also critical contributors (16, 17). Additionally, lifestyle factors such as body mass index (BMI), physical activity levels, and ergonomic challenges during pregnancy and postpartum, such as improper lifting techniques or prolonged periods of standing, may influence the onset or severity of LBP (6, 16, 18).

Cesarean sections (C/S) are now increasingly common worldwide, with rates rising in both developed and developing countries. While C/S are necessary in many cases, they come with increased morbidity and complications, including post-surgical LBP. Spinal anesthesia is commonly used during C/S and is associated with post-spinal low back pain in 13%−44.9% of cases (19, 20). This pain can be caused by factors such as spine immobilization, repeated dural puncture by the spinal needle, prolonged surgery, high body mass index, and muscle relaxation (21). Post-dural puncture pain is a specific type of localized pain that occurs at the site where the spinal needle is inserted, often due to para-spinal muscle relaxation and inflammation (19, 22).

The physical act of labor and pushing during spontaneous vaginal delivery (SVD) places considerable pressure on the lower back and pelvic area (7). Factors related to labor, such as the duration and intensity of labor, the use of anesthesia (particularly epidural), and the mother's positioning during delivery, can affect the risk of developing low back pain (13, 18, 19). Hormonal changes that cause ligamentous laxity during pregnancy may persist after childbirth, making the spine more prone to strain and injury. Inadequate postpartum recovery can also worsen back pain when women resume physical activities too quickly or without proper support (23).

Addressing postpartum low back pain is crucial for promoting the wellbeing of mothers. Early diagnosis, proper management, and targeted interventions can not only alleviate pain but also improve a mother's physical function, emotional state, and overall quality of life during this critical time of recovery and transition. This study aimed to assess the incidence of low back pain following cesarean delivery and normal vaginal delivery, hypothesizing that mothers who deliver via cesarean section have a higher incidence of low back pain. Additionally, it sought to identify the risk factors associated with this low back pain and evaluate its impact on the quality of life after delivery. By addressing these factors, healthcare providers can enhance postpartum care and improve the wellbeing of new mothers.

## Methods and materials

### Study setting and population

A prospective cohort study was conducted at Dilla University General Hospital (DUGH) from November 2021 to June 2023. DUGH is one of the largest governmental teaching hospitals in Ethiopia's southern region, specifically in Gedeo Zone, Dilla Town. It is ~360 km from Addis Ababa on the main road to Nairobi and about 411 km from the Ethiopia-Kenya border in the southern part of Ethiopia.

We attempted to perform this study following the STROBE guidelines for observational studies (https://www.strobe-statement.org). This study was also registered on the research registry with the unique identification number of researchregistr10668 (https://www.researchregistry.com/browse-the-registry#home/). All pregnant mothers classified as American Society of Anesthesiology (ASA) II and III, who presented themselves for delivery at DUGH and volunteered to participate in the study during the data collection period, were included. However, patients with pre-existing back pain or those with communication difficulties after surgery (such as disabilities, lack of phone access, or residing outside the DUGH child vaccination area) were excluded.

### Operational definitions

#### American Society of Anesthesiologists (ASA)

Physical Status Classification System is a risk-stratifying system used to assess a patient's physical status for stratification and optimization of patients before surgery (24).

#### Low back pain

Low back pain is a pain or stiffness in the lower back characterized by localization to lumbar and lower thoracic regions, a continuous type of pain that sometimes radiates to legs.

### Sample size and sampling technique

The sample size is calculated using a two-population formula after conducting a pilot study on 22 pregnant mothers. The incidence of low back pain on the seventh day was used, as it provides the maximum required sample size. The results showed an incidence of 44.9% in the C/S group and 21.6% in the SVD group. With a 95% confidence interval and 80% power, the sample size for one group is 61. To accommodate a 10% nonresponse rate, a total of 134 participants were selected using a systematic random sampling technique.

### Data quality management and data collection method

Ethical clearance was obtained from the Institutional Review Board (IRB) of Dilla University College of Medicine and Health Science with the reference number duirb/020/22-01. Written informed consent was obtained from each study participant. Data were collected by chart review and patient interviews through patient visits before discharge and phone calls or on their arrival for child vaccination after discharge from the hospital, using pretested structured questionnaires by trained data collectors. Supervisors and the principal investigator provided daily supervision throughout the data collection period. Participants were grouped based on their exposure to cesarean section (CS group) or spontaneous vaginal delivery (SVD group), with a 1:1 ratio. Postpartum data on the presence and severity of back pain were collected at various intervals: within the first 24 h; on the 2nd, 3rd, 4th, 5th, 6th, and 7th days; on the 2nd, 3rd, and 4th weeks; and the 2nd, 3rd, 4th, 5th, and 6th months. The severity of back pain was measured using the Numerical Rating Scale score.

The Short-Form 36 (SF-36) version 2 was used to assess the quality of life. This tool has been translated into Amharic and validated for use in Ethiopia, based on a study conducted in Butajira, rural Ethiopia. The Cronbach's alpha for most components exceeded 0.70 except for the vitality domain, which recorded a Cronbach's alpha of 0.68 (25). The SF-36 measures both the physical and mental components of quality of life comprising 36 items distributed across eight domains: physical functioning (10 items), role limitations due to physical health (four items), bodily pain (two items), social functioning (two items), general mental health (psychological distress and wellbeing) (five items), role limitations due to emotional problems (three items), vitality (energy/fatigue) (four items), and general health perceptions (five items). Each domain is scored from 0 (indicating the worst possible state) to 100 (indicating the best possible state). Data on quality of life were collected during visits for 6-month child vaccinations or through phone calls during the 6-month postpartum period. In cases where respondents could not come for vaccination or their phones were not working, data collectors made additional attempts to reach them up to five times. Higher scores indicate a better quality of life, while lower scores reflect an impaired quality of life.

### Data analysis and interpretation

After the completion of data collection, the data were manually checked for completeness, coded, and entered into SPSS version 25 software for analysis. Descriptive statistics, such as frequencies, proportions, means, and standard deviations, were used for reporting. The chi-square test was used to analyze categorical data, while the Student's *t*-test was used for continuous data. Effect sizes were calculated to determine the magnitude of the differences. Bivariate and multivariate logistic regression analyses were performed to explore the relationships between dependent and independent variables. Variables with a *p*-value of < 0.25 in the bivariate analysis were included in the multivariate logistic regression to control for confounding factors. Associations between variables were evaluated using crude odds ratios (COR) and adjusted odds ratios (AOR) with 95% confidence intervals. Statistical significance was set at a *p*-value of < 0.05.

## Results

**Figure 1:**
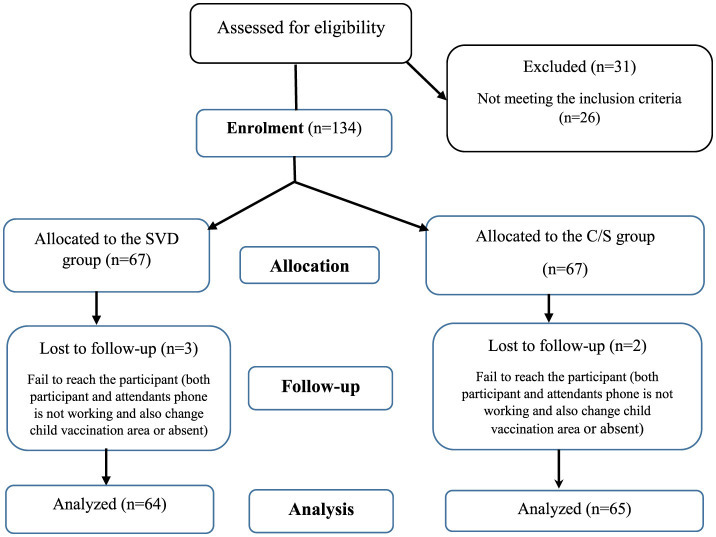
STROBE flow diagram of the study. C/S, cesarean section; SVD, spontaneous vaginal delivery; *n*, frequency.

A total of 134 women who met the inclusion criteria and provided informed consent were selected for the study. This cohort comprised 67 women who underwent cesarean sections (C/S) and 67 women who had spontaneous vaginal deliveries (SVD). However, two participants from the C/S group and three from the SVD group were lost to follow-up due to unsuccessful attempts to contact them. Ultimately, 65 participants from the C/S group and 64 from the SVD group completed the follow-up, resulting in a total of 129 participants (96.3%) included in the final analysis (Figure 1).

### Demographic and obstetrics characters

There were no significant differences between the groups regarding age, ASA classification, body mass index, gestational age, gravidity, or parity. The majority of participants in both groups were aged between 26 and 32 years, classified as ASA II, and had reached term gestational age. Furthermore, most of the participants were multiparous (Table 1).

**Table 1:** Socio-demographical and obstetrics characteristics of pregnant women delivered by C/S and SVD in Dilla University General Hospitals.

**Variable**	**Category**	**C/S *N* (%)**	**SVD *N* (%)**	***P*-value**
Age in year	< 25	25 (38.5%)	20 (31.3%)	0.140
	26–32	32 (49.2%)	41 (64.1%)	
	>33	8 (12.3%)	3 (4.7%)	
ASA	ASA II	60 (92.3%)	62 (96.9%)	0.252
	ASA III	5 (7.7%)	2 (3.1%)	
BMI	18.5–24.9	29 (44.6%)	37 (57.8%)	0.156
	24.5–29.9	26 (40%)	23 (35.9%)	
	30–34.9	10 (15.4%)	4 (6.3%)	
Gestational age	< 37	8 (12.3%)	7 (10.9%)	0.262
	37–42	46 (70.5%)	52 (81.3%)	
	>42	11 (16.9%)	5 (7.8%)	
Gravidity	Primigravida	17 (26.1%)	11 (17.2%)	0.217
	Multigravida	48 (73.9%)	53 (82.8%)	
Parity	Primiparous	19 (29.2%)	14 (21.9%)	0.338
	Multiparous	46 (70.8%)	50 (78.1%)	

ASA, American Society of Anesthesiology Classification; BMI, body mass index; C/S, cesarean section; SVD, spontaneous vaginal delivery; N, frequency.

### Incidence of low back pain

**Figure 2:**
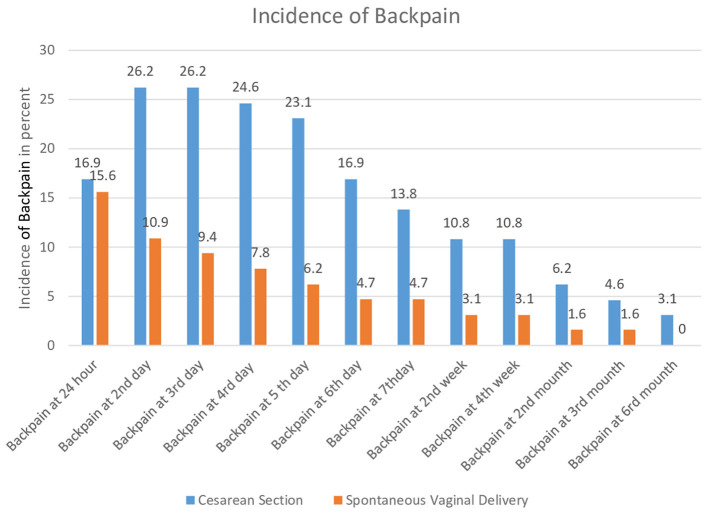
Incidence of low back pain in women with spontaneous vaginal delivery and cesarean section at Dilla University.

The incidence of low back pain in the C/S group was 26.2% on the second postpartum day, 13.8% at 7 days postpartum, and 10.8% at 4 weeks postpartum. In contrast, the SVD group reported incidences of 10.9% on the second postpartum day, 4.7% at 7 days postpartum, and 3.1% at 4 weeks postpartum. The C/S group exhibited a significantly higher incidence of low back pain from the second day up to 4 weeks postpartum compared to the SVD group, with a *p*-value of < 0.05. However, no significant differences were observed in the incidence of low back pain between the two groups on the first postpartum day or after 1 month (Figure 2, Table 2).

**Table 2:** Severity low back pain of pregnant women delivered by C/S and SVD in Dilla University General Hospitals.

**Variables**	**C/S (65 mothers)**			**SVD (64 mothers)**			***P*-value**
	**Severe (** ***N*** **)**	**Moderate (** ***N*** **)**	**Mild (** ***N*** **)**	**Severe (** ***N*** **)**	**Moderate (** ***N*** **)**	**Mild (** ***N*** **)**	
Back pain at 24 h	1	3	7	–	3	7	0.88
Back pain at 2nd day	1	4	12	–	1	6	0.013^*^
Back pain at 3rd day	1	3	14	–	–	6	0.0014^*^
Back pain at 4th day	–	2	14	–	–	5	0.002^*^
Back pain at 5th day	–	1	14	–	–	3	0.0015^*^
Back pain at 6th day	–	1	10	–	–	2	0.015^*^
Back pain at 7th day	–	1	8	–	–	2	0.02^*^
Back pain at 2nd week	–	–	7	–	–	2	0.043^*^
Back pain at 3rd week	–	–	7	–	–	2	0.043^*^
Back pain at 4th week	–	–	7	–	–	2	0.043^*^
Back pain at 2nd month	–	–	4	–	–	1	0.06
Back pain at 3rd month	–	–	3	–	–	1	0.11
Back pain at 4th month	–	–	3	–	–	–	0.06
Back pain at 5th month	–	–	2	–	–	–	0.11
Back pain at 6th month	–	–	2	–	–	–	0.11

N, frequency; C/S, cesarean section; SVD, spontaneous vaginal delivery.

^*^Statistically significant difference at p < 0.05 between the C/S and SVD group.

### Severity of low back pain

Most participants in both groups experienced mild lower back pain during the follow-up period. Among the C/S group, 4 out of 17 participants reported moderate pain on the second postpartum day, but no moderate pain was reported after the seventh postpartum day. In the SVD group, only one out of 10 participants reported experiencing moderate pain on the second postpartum day, with no moderate pain reported thereafter. Severe low back pain was reported by only one mother in the C/S group during the first 3 days, while no severe pain was reported in the SVD group (Table 2).

### Risk factors associated with low back pain

In the bivariate analysis, factors such as maternal age, gestational age, parity, and body mass index (BMI) were found to be associated with post-delivery low back pain, with *p*-values < 0.25, which indicated that they were potential candidates for inclusion in the multiple logistic regression analysis. After adjusting for other confounders, it was discovered that mothers with a body mass index >30 kg/m^2^ had a significantly higher risk of experiencing post-delivery low back pain [AOR = 3.01 (1.92–5.43), *p* = 0.013]. Additionally, mothers with a post-term gestational age were also found to have an increased risk of post-delivery low back pain [AOR = 1.79 (1.23–7.75), *p* = 0.025; Table 3].

**Table 3:** Risk factors associated with low back pain after delivery.

**Variables**	**Categories**	**Low back pain**		**COR**	**AOR**	***P*-value**
		**Yes (** ***N*** **)**	**No (** ***N*** **)**			
Age	< 25 years	7	38	1	1	0.180 0.067
	26–32 years	16	57	1.52 (0.57–4.05)	1.92 (0.63–5.86)	
	>33 years	4	7	3.1 (0.71–13.46)	3.49 (0.66–18.35)	
BMI	18.5–24.9 kg/m^2^	8	58	1	1	0.084 0.013^*^
	24.5–29.9 kg/m^2^	13	36	2.62 (0.99–6.93)	2.92 (0.93–7.48)	
	>30 kg/m^2^	6	8	5.44 (1.49–19.77)	3.01 (1.28–9.94)	
Parity	Primiparous	4	29	1	1	0.137
	Multiparous	23	73	2.28 (0.73–7.18)	1.88 (0.52–6.73)	
Gestational age	< 37	2	13	1	1	0.132 0.025^*^
	37–42	18	80	1.46 (0.30–7.05)	1.29 (0.25–6.67)	
	>42	7	9	5.05 (0.84–30.17)	1.79 (1.13–53.96)	

N, frequency; BMI, body mass index; COR, crude odd ratio; AOR, adjusted odd ratio.

^*^ and bold indicate statistically significant at *p* < 0.05.

1 = reference.

### Quality of life in mothers with low back pain after delivery

A statistically significant difference was observed between the groups in terms of health-related quality of life. Mothers who delivered vaginally and experienced back pain had a higher score for health-related quality of life (79.13 ± 7.06) compared to those who delivered via C/S and experienced back pain (73.12 ± 3.46), with a *p*-value of 0.006 and an effect size of 0.48 (Table 4).

**Table 4:** Quality of life associated with low back pain after delivery.

**SF-36 HRQoL**		**C/S with low back pain (mean ±SD)**	**SVD with low back pain (mean ±SD)**	***P*-value**
**Component**	**Number of items**			
Physical functioning	10	72.11 ± 8.85	80.95 ± 5.86	0.01^*^
Role limitations due to physical health	4	67.01 ± 11.8	75.92 ± 8.78	0.048^*^
Bodily pain	2	72.72 ± 8.22	80.14 ± 4.11	0.014^*^
General health	5	67.11 ± 14.26	82.46 ± 5.43	0.003^*^
Social functioning	2	74.71 ± 7.77	80.26 ± 7.20	0.082^*^
Emotional wellbeing	5	77.24 ± 5.53	78.26 ± 7.59	0.689
Role limitations due to emotional problems	3	75.95 ± 10.77	77.79 ± 10.10	0.666
Vitality	4	68.01 ± 12.28	77.27 ± 4.60	0.032^*^
Total		71.86 ± 7.06	79.13 ± 3.46	**0.006** ^*^

HRQoL, health related quality of life; C/S, cesarean section; SVD, spontaneous vaginal delivery; SD, standard deviation.

^*^ and bold indicate statistically significant at *p* < 0.05.

## Discussions

This study found that the incidence of LBP was significantly higher in the C/S group compared to the SVD group during the second postpartum day up to the fourth week. On the second postpartum day, 26.2% of mothers in the C/S group experienced LBP, compared to 10.9% in the SVD group (*p*-value = 0.013). By the fifth day, the incidence dropped to 23.1% in the C/S group and 6.2% in the SVD group (*p*-value = 0.0015). By the seventh day, the incidence decreased further to 13.8% in the C/S group and 4.7% in the SVD group (*p*-value = 0.02). By 4 weeks postpartum, the incidence had decreased even more to 10.8% in the C/S group and 3.1% in the SVD group (*p*-value = 0.043). These findings indicate that while LBP is more common in the immediate postpartum period among mothers who underwent a C-section with spinal anesthesia, this might be due to the effects of spinal anesthesia, surgical trauma, and extended immobility following the procedure. Additionally, mothers who delivered vaginally at this hospital did not receive epidural anesthesia for pain relief.

The findings of our study were consistent with findings from previous studies that suggested C/S is associated with a higher risk of postpartum LBP. For instance, research done by Chia et al. (21) found that women who underwent C/S were more likely to experience persistent back pain due to factors like epidural or spinal anesthesia, surgical trauma, prolonged immobility post-surgery, and challenges of postoperative recovery. On the other hand, our results were in contrast with some studies (11, 20) which found that there is no significant difference in postpartum LBP between C/S and SVD groups after the immediate postpartum period. This discrepancy could be due to variations in study populations, pain assessment methods, differences in postpartum care practices, or the extent of physical activity during recovery contributing to the differing results.

Even though the incidence of low back pain was not statistically significant after the first month postpartum, the C/S group experienced higher rates of LBP at 2, 3, and 6 months compared to the SVD group. Specifically, the C/S group had incidences of LBP 6.2% at 2 months, 4.6% at 3 months, and 3.1% at 6 months. In contrast, the SVD group consistently had a low incidence of 1.6% at both 2 and 3 months, with no reports of LBP at 6 months. These findings suggest that mothers who underwent C/S may experience a longer recovery period for LBP due to the lasting effects of surgery and anesthesia, such as prolonged tissue healing and altered biomechanics. Therefore, long-term monitoring and targeted interventions for persistent LBP in C/S mothers are crucial beyond the immediate postpartum period.

Our study revealed that mothers who delivered via C/S under spinal anesthesia experienced a higher intensity of LBP in the first postpartum period compared to those who had SVD. However, this pain significantly decreased after the first week, suggesting that the initial postoperative period is critical for pain management. This finding aligns with preexisting literature (11, 12, 26) that noted LBP intensity is often highest during the first week after C/S due to the immediate physical trauma of surgery and early wound healing, particularly in the early postpartum period. As the body recovers and mothers adapt to their new physical state, the pain typically decreases in intensity. This emphasizes the importance of implementing effective pain management strategies during this critical period to alleviate discomfort.

Our study also showed that a BMI >30 kg/m^2^ and postdate gestational age have an increased risk of LBP. This study was in line with the study done by Breen et al. (27) which found that overweight and obese women are at a greater risk of developing LBP due to the increased mechanical load on the spine and the associated inflammatory processes. Similarly, the link between post-term gestation and LBP may be related to the prolonged physical strain on the mother's body, as well as the potential for larger fetal size, which can contribute to musculoskeletal discomfort during and after delivery.

This study showed a statistically significant difference in health-related quality of life between the two groups. Mothers who delivered via SVD and experienced LBP had a higher health-related quality of life score (79.13 ± 7.06) compared to those who delivered via C/S and experienced back pain (73.12 ± 3.46), with a *p*-value of 0.006 and an effect size of 0.48. This finding highlights the potential long-term impact of delivery mode and LBP on maternal wellbeing, with vaginal delivery being associated with better postpartum health-related quality of life outcomes.

This observation aligns with the findings of most studies (15, 28, 29), which reported that women who undergo C/S often face longer recovery times and greater physical limitations, which can negatively impact their quality of life. The reduced quality of life in the C/S group may be attributed to a combination of factors, including prolonged pain, slower recovery, and the psychological impact of undergoing major surgery. These results underline the importance of providing comprehensive postpartum care that addresses both the physical and psychological aspects of recovery, particularly for mothers who have undergone C/S.

Assessing LBP following C/S and SVD is vital for guiding clinical practice and advancing research. Understanding the incidence, severity, and associated factors of postpartum back pain is essential for developing targeted pain management strategies, optimizing recovery, and improving maternal health outcomes. Identifying risk factors such as overweight status, post-term gestational age, and pre-existing conditions facilitates early interventions to prevent chronic pain. From a research perspective, examining the differences in pain outcomes between delivery methods provides insights into the biomechanical, hormonal, and procedural effects on maternal health. Evaluating the impact of postpartum back pain on quality of life highlights the need for comprehensive postpartum care that addresses physical, emotional, and functional wellbeing, thereby informing policies and interventions that promote long-term health for mothers.

A limitation of our study is the exclusion of mothers with communication difficulties, such as those without phone access, those whose children were vaccinated outside our study area, or those with disabilities affecting communication. As a result, our findings may not be inferred for rural populations, where phone access is limited. Another limitation is that the C/S group did not include women who received epidural anesthesia. Similarly, no women in the SVD group received spinal or epidural anesthesia, as the use of epidural anesthesia is not routinely practiced in our hospital. To gain a more comprehensive understanding, future research could consider including these populations by conducting house-to-house data collection with the assistance of local healthcare professionals.

## Conclusions

This study contributes to the existing evidence, which suggests that Cesarean delivery is associated with a higher incidence and more severe low back pain after childbirth compared to natural vaginal delivery. It identifies specific risk factors such as higher BMI and post-term gestation, providing valuable information for targeted interventions. Moreover, the study recommends the significance of comprehensive postpartum care, including pain management and support for affected mothers, by emphasizing the impact of low back pain on quality of life. Further research could explore long-term outcomes and evaluate the efficacy of different pain management strategies for this particular group.

## Data Availability

The raw data supporting the conclusions of this article will be made available by the authors, without undue reservation.
